# *Polygonatum cyrtonema* Hua in Jiuhua Mountain: Structural Characterization and Biological Activity

**DOI:** 10.3390/molecules31193542

**Published:** 2026-10-04

**Authors:** Xueqing Geng, Xingchi Zhang, Yun Wang, Ting Yao, Zhuoting Gan

**Affiliations:** 1 School of Bioengineering, Huainan Normal University, Huainan 232038, China; 2 Key Laboratory of Bioresource and Environmental Biotechnology of Anhui Higher Education Institutes, Huainan Normal University, Huainan 232038, China; 3 College of Life and Environmental Sciences, Huangshan University, Huangshan 245700, China; 4 School of Tourism, Huangshan University, Huangshan 245700, China

**Keywords:** *Polygonatum cyrtonema* Hua in Jiuhua, polysaccharide, structural characterization

## Abstract

*Polygonatum cyrtonema* Hua in Jiuhua Mountain (JHPCH) is a nationally recognized geographically indicated medicinal plant known for its bioactive polysaccharides with diverse pharmacological activities. This study isolated a homogeneous polysaccharide JPCP-70-0 from JHPCH via hot water extraction, ethanol precipitation, and chromatographic purification. Structural characterization by GC-MS, FT-IR, and NMR identified it as a neutral fructan (2.88 × 10^4^ Da) with Glc/Fru (6.65%/93.35%), a backbone of →1)-β-D-Fru*f*-(2→, and side chains of →6)-β-D-Fru*f*-(2→). Using Caenorhabditis elegans as a model, we evaluated JPCP-70-0’s effects on nematode growth. Results showed concentration-dependent regulation: low concentrations promoted growth, locomotion, feeding, and reproduction, while high concentrations induced developmental inhibition and reduced activity. This work provides a theoretical basis for the rational development of JHPCH resources.

## 1. Introduction

Natural products derived from medicinal plants serve as an important reservoir of bioactive compounds, thus occupying an irreplaceable position in traditional medical systems and modern drug development. Among numerous bioactive components, polysaccharides have become a research hotspot in the fields of medicine and nutrition in recent years due to their diverse biological activities (e.g., antioxidant, immunomodulatory, anti-inflammatory, and anti-aging effects [1,2,3,4]), as well as their low toxicity and high biocompatibility [5,6]. Plant polysaccharides are major bioactive components, with their structural diversity directly determining their functional properties [7,8]. Thus, the exploration of their extraction and purification, structural characterization, and structure–activity relationships has been identified as the core research direction at present.

The dried rhizome of *Polygonatum cyrtonema* belonging to the Liliaceae family is recorded in the Chinese Pharmacopoeia to have three botanical origins: *Polygonatum sibiricum* Red., *Polygonatum kingianum* Coll.et Hemsl., and *Polygonatum cyrtonema* Hua. *Polygonatum cyrtonema* contains various nutritional and bioactive components, exhibiting multiple pharmacological activities, including anti-aging, anti-inflammatory, hypoglycemic, and memory-improving effects [9,10,11,12,13]. Among these components, polysaccharides are recognized as the major bioactive constituents of *Polygonatum cyrtonema* and are designated as the quality marker in the Chinese Pharmacopoeia [14]. Given the edible and medicinal homology characteristics of *Polygonatum cyrtonema* polysaccharides, the structural characterization and functional exploration of these natural polysaccharides have become a prevailing research focus. In general, the structural characteristics of plant polysaccharides are mainly characterized by monosaccharide composition, glycosidic linkage configuration and connection mode. Previous studies have systematically revealed the structural features of *Polygonatum* polysaccharides. Gao et al. [15] characterized the purified polysaccharide fraction PHP-1 from *Polygonatum cyrtonema* Hua, verifying that PHP-1 is a branched fructan with a peak molecular weight of 4.56 × 10^3^ Da. Methylation analysis demonstrated that PHP-1 is dominated by fructofuranosyl residues including →1)-β-D-Fru*f*-(2→, β-D-Fru*f*-(2→, →1,6)-β-D-Fru*f-*(2→ and →6)-β-D-Fru*f*-(2→, together with a small proportion of →6)-α-D-Glc*p*-(1→ residues; the →1)-β-D-Fru*f*-(2→ units form the primary backbone, which is substituted at the O-6 position by short side-chains of β-D-Fru*f*-(2→ and →6)-β-D-Fru*f*-(2→ residues. Additionally, Li et al. [16] conducted structural analysis on *Polygonatum kingianum* polysaccharide PKPs-1, and the methylation results revealed that PKPs-1 is composed of five monosaccharide derivatives, namely T-Glc*p*, 1,4-linked Glc*p*, 1,6-linked Glc*p*, 1,2-linked Glc*p*, and 1,2-linked Man*p*, with a molar ratio of 1.12:0.78:2.52:3.58:2.00. The backbone of PKPs-1 is formed by glucose residues, while its branched structures are constructed by 1→2, 1→4, 1→6-linked glucose and 1→2-linked mannose with repetitive branching characteristics. These studies have laid a solid structural foundation for exploring the biological functions of *Polygonatum* polysaccharides. Wang et al. [17] characterized the purified polysaccharide fraction PCP-J-1 from *Polygonatum cyrtonema* Hua, verifying that PCP-J-1 is a branched fructan with an average molecular weight of 7.384 kDa. Methylation analysis demonstrated that PCP-J-1 consists of →1)-β-D-Fru*f*-(2→, →1,6)-β-D-Fru*f*-(2→, β-D-Fru*f*-(2→ and trace →6)-α-D-Glc*p*-(1→ residues, among which →1)-β-D-Fru*f*-(2→ and →1,6)-β-D-Fru*f*-(2→ residues constitute the main backbone structure.

Significant differences are observed in the chemical composition and biological activities of *Polygonatum cyrtonema* polysaccharides, with variations in species, production areas, and extraction methods. *Polygonatum cyrtonema* Hua in Jiuhua Mountain (JHPCH) refers to the species growing in Jiuhua Mountain and its surrounding areas. It is also named Dizang Huangjing, owing to the unique natural environment and Buddhist culture. It is renowned for its distinctive medicinal and tonic effects, and hence is listed as one of the “top ten Anhui medicinal herbs” of Anhui Province. It was approved as a nationally protected geographical indication product in 2017.

JHPCH contains various components, including polysaccharides, steroidal saponins, alkaloids, flavonoids, and amino acids. As a typical medicinal-and-edible plant, it is mainly administrated via oral route. However, raw JHPCH may cause throat irritation and a numbing tongue sensation, so processed products are preferred for medicinal applications. The nine-time steaming and nine-time sun-drying method is the major processing approach for raw JHPCH, which can alleviate its irritant effects to facilitate oral consumption and simultaneously boost its pharmacological potency. JHPCH polysaccharides (JHPCPs) are one of the main active components, and also the only component specified for content determination in the Chinese Pharmacopoeia. A large number of studies have been conducted on JHPCPs in recent years, with continuous enhancement in research depth. Modern pharmacological studies have confirmed the antioxidant, anticoagulant, antitumor, and significant immunomodulatory effects of JHPCPs, indicating that water-soluble polysaccharides are one of the major bioactive constituents responsible for its pharmacological activities [13,14,18,19].

*Caenorhabditis elegans* (*C. elegans*), a small soil-dwelling nematode feeding on bacteria, is widely employed as a model organism in the fields of toxicology, disease modeling, genetics and development, locomotion and behavior, and others [20], owing to its easy cultivation, a short life cycle, low experimental cost, high fecundity, a simple anatomical structure, and simple model establishment. The growth and development of *C. elegans* have been explored across a broad spectrum of fields, ranging from organ development to antibacterial drug screening, nanotoxicology model construction, lifespan-extending effects and their underlying mechanisms, the regulatory effects of inactivated *Escherichia coli* on nematode development [21], the developmental function of CT14, and neural regulation of innate immune function. To date, few studies have examined the effects of JHPCPs on the growth and development of *C. elegans*.

This study aimed to systematically investigate the extraction, purification, structural characterization, and bioactivity of JHPCPs. Its specific objectives were as follows: (1) extracting JHPCPs via the water extraction and ethanol precipitation method to obtain purified polysaccharide fractions; (2) characterizing the structural characteristics using advanced analytical techniques including high-performance liquid chromatography (HPLC), gas chromatography–mass spectrometry (GC-MS), Fourier-transform infrared (FT-IR) spectroscopy, and nuclear magnetic resonance (NMR); (3) exploring the effects of JHPCPs on the growth and development of *C. elegans*; and (4) elucidating the response mechanism to different concentrations of the polysaccharides, so as to lay a theoretical foundation for the development of anti-aging preparations based on JHPCPs.

## 2. Experimental

### 2.1. Materials

*Polygonatum cyrtonema* was collected from Jiuhua Mountain, Xuancheng City, Anhui Province, China. DEAE-52 cellulose and Sephadex G-100 were purchased from Beijing RuiDaHengHui Science & Technology Development Co., Ltd., Beijing, China. Deuterium oxide (D_2_O) was purchased from Shanghai Aladdin Biochemical Technology Co., Ltd., Shanghai, China. Dextran standards, monosaccharide standards, and dimethyl sulfoxide (DMSO) were purchased from Shanghai Macklin Biochemical Co., Ltd., Shanghai, China.

### 2.2. Extraction, Separation, and Purification of Polysaccharides

Briefly, JHPCH was sliced and dried in a dryer at 60 °C. The dried slices were pulverized into powder using a grinder and stored in a desiccator for subsequent use. For extraction, 100 g of the obtained powder was used with a material-to-liquid ratio set at 1:20 (g:mL), and extraction was performed via two cycles at 80 °C for 2 h. The combined filtrate was concentrated to 20% of the original volume, followed by precipitation with 80% (*v*/*v*) ethanol for 12 h. The precipitate was collected after centrifugation (20 min, 4000 rpm) and then fully dissolved in distilled water. The organic phase was removed by rotary evaporation (RE-52AA, Shanghai Yarong Biochemical Instrument Factory, Shanghai, China), thereby obtaining the crude polysaccharide extract. Deproteinization and decolorization of the crude polysaccharide extract were performed by the Sevag method and the hydrogen peroxide method, respectively. The solution was dialyzed against running water for 3 days, and the crude polysaccharide (designated JPCP) was obtained after freeze-drying by lyophilizer (Model 25EL, Shanghai Bilang Instrument Manufacturing Co., Ltd., Shanghai, China). JPCP was dissolved in deionized water (10 mg/mL), and preliminary purification was conducted by graded ethanol precipitation (20%, 50%, and 70%, *v*/*v*).

JPCP-70 was dissolved in deionized water (50 mg/mL) and filtered through a 0.45 μm membrane filter. The filtrate was loaded onto a DEAE-52 anion-exchange column preequilibrated with five column volumes of buffer. Gradient elution was performed using NaCl solutions at concentrations of 0, 0.1, 0.3, 0.5, and 0.7 mol/L, with an elution flow rate of 6 mL per tube controlled with a constant flow pump. Fractions corresponding to the target elution peak were pooled, dialyzed for 72 h (3500 Da), and lyophilized under vacuum to yield the preliminarily purified polysaccharide. The obtained polysaccharide was redissolved in deionized water at a concentration of 20 mg/mL, filtered through a 0.45 μm membrane filter, and then applied to a Sephadex G-100 gel column. The elution rate was controlled at 2 mL per tube using the constant-flow pump. Tubes with the highest absorbance values were collected and lyophilized under vacuum to obtain the purified polysaccharide, designated as JPCP-70-0.

### 2.3. Structural Identification

#### 2.3.1. Analysis of Molecular Weight Distribution

Dextran standards and JPCP-70-0 (1 mg/mL) were accurately weighed and analyzed using HPLC [22] (Agilent Technologies (China) Co., Ltd., Beijing, China) equipped with a TSK-GEL G4000 PWXL column (7.8 mm × 300 mm) and refractive index detector (RID). The refractive index detector was used for determining elution times. The chromatographic parameters were set as follows: column temperature, 25 °C; injection volume, 20 μL; ultrapure water, mobile phase; flow rate, 1.0 mL/min; and run time, 25 min. A standard calibration curve was constructed with retention time as the abscissa and the logarithm of molecular weight as the ordinate. The molecular weight of JPCP-70-0 was calculated based on the established calibration curve.

#### 2.3.2. Determination of Basic Components of JPCP-70-0

The total carbohydrate content was determined by the phenol–sulfuric acid method [23]. The protein content was measured by the Coomassie brilliant blue method [24]. The uronic acid content was quantified using the *m*-hydroxybiphenyl method [25].

#### 2.3.3. Determination of Monosaccharide Composition of JPCP-70-0

The monosaccharide composition of JPCP-70-0 was determined using a high-performance ion chromatograph (ICS 5000+; Thermo Fisher Scientific (China) Co., Ltd., Shanghai, China) coupled with an electrochemical detector [26]. JPCP-70-0 and 13 monosaccharide standards (Fuc, Rha, Ara, Gal, Glc, Xyl, Man, Fru, Rib, Gal-UA, Gul-UA, Glc-UA, and Man-UA) were accurately weighed. Each sample was hydrolyzed with 1 mL of TFA (2 mol/L) at 60 °C for 1 h. The hydrolysate was dried under a stream of nitrogen gas. The residue was rinsed with 99.99% methanol and dried again, and this washing procedure was repeated two to three times. The dried residue was reconstituted with sterile water and transferred into a chromatography vial for subsequent analysis.

The chromatographic conditions were set as follows: Dionex Carbopac™ PA20 column (Thermo Fisher Scientific (China) Co., Ltd., Shanghai, China) (150 × 3.0 mm^2^, 10 μm); ternary gradient elution (A: H_2_O; B: 0.1 mol/L NaOH; C: 0.1 mol/L NaOH + 0.2 mol/L NaAc); flow rate, 0.5 mL/min; injection volume, 5 μL; column temperature, 30 °C. Detailed elution parameters are shown in Supplementary Table S1.

#### 2.3.4. Analysis of Chemical Groups

For analyzing chemical groups, 1 mg of JPCP-70-0 was ground with 100 mg of anhydrous KBr and then compressed into a pellet. Using KBr as the blank reference, the infrared spectrum was recorded on an FT-IR spectrometer (IS-50; Nicolet Instrument Corporation, Waltham, MA, USA).

#### 2.3.5. Methylation Determination of JPCP-70-0

After methylation, hydrolysis, and acetylation, the glycosidic linkage patterns of JPCP-70-0 were determined using GC-MS combined with comparison against a standard mass spectral library [26]. JPCP-70-0 (5 mg) was dissolved in DMSO with ultrasonic assistance, followed by the sequential addition of methylation reagents A and B (NaOH–DMSO and iodomethane). The reaction system was purged with nitrogen gas and incubated in the dark for 1 h. Next, 250 μL of iodomethane was added, and the reaction was continued for another 1 h. The methylation reaction was terminated by adding 2 mL of ddH_2_O. The mixture was dialyzed for 24 h and then lyophilized. The fully methylated sample was hydrolyzed with 1 mL of TFA (2 mol/L, 120 °C, 90 min), followed by evaporation to dryness under a nitrogen stream. The residue was redissolved in 2 mL of ddH_2_O and reduced with NaBH_4_ (30 mg) at room temperature for 12 h. After neutralization with CH_3_COOH, the solution was dried in an oven at 105 °C. Acetylation was performed by adding C_4_H_6_O (1 mL, 100 °C, 1 h), and the acetylation reaction was quenched with ultrapure water after cooling. The product was extracted with 3 mL of CH_2_Cl_2_ and washed with distilled water four times. The CH_2_Cl_2_ layer was dried over Na_2_SO_4_, concentrated to 1 mL, filtered through a 0.22 μm organic membrane filter, and subjected to GC-MS analysis.

GC-MS analysis was performed using an HP-5MS capillary column (30 m × 0.25 mm × 0.25 μm; Agilent J&W Scientific, Folsom, CA, USA). The temperatures of the injection port and the detector were set to 260 °C, whereas the He flow rate was maintained at 1 mL/min. For temperature programming, the initial temperature was held at 50 °C for 1.0 min, raised to 130 °C at a rate of 50 °C/min, further increased to 230 °C at a rate of 3 °C/min, and then held for 2 min.

#### 2.3.6. Nuclear Magnetic Resonance Analysis

JPCP-70-0 (50 mg) was dissolved in 1 mL of D_2_O, and 1D and 2D NMR analyses (^1^H-NMR, ^13^C-NMR, ^1^H-^1^H COSY, HSQC, HSQC-TOCSY, HMBC, and NOESY) were performed at 25 °C using a Bruker AVANCE III HD 600 MHz spectrometer (Bruker Biospin GmbH, Rheinstetten, Germany). Chemical shifts were calibrated as follows: the residual HDO signal was set at *δ*_h_ = 4.70 ppm, and the methyl carbon signal of tetramethylsilane was referenced to *δ*C = 0.00 ppm.

### 2.4. The Impact of JPCP-70-0 on the Growth and Development of C. elegans

#### 2.4.1. Experimental Materials and *C. elegans* Cultivation

Wild-type *C. elegans* (N2 strain) and *E. coli* OP50 were preserved in our laboratory. Nematodes were cultured routinely on NGM seeded with OP50 in a constant-temperature incubator at 20 °C. A synchronized population was obtained by collecting eggs using the NaOCl–NaOH lysis method, transferring them to bacteria-free NGM plates, and incubating them for 16–18 h to yield synchronized larvae arrested in the L1 stage [27].

#### 2.4.2. Drug Administration and Experimental Grouping

JPCP-70-0 was dissolved in sterile Km buffer to prepare a stock solution. The stock solution was mixed with *E. coli* culture in proportional volumes to prepare NGM plates supplemented with a serial concentration gradient of JPCP-70-0 (0 ng/mL, 2 ng/mL, and 500 mg/mL). Synchronized L1-stage larvae were transferred onto NGM plates containing the corresponding polysaccharide concentrations for treatment until all indicators were measured.

#### 2.4.3. Measurement of Development and Morphological Indicators

Tolerance and survival rate: The survival and developmental timeline of nematodes from the L1 stage to the gravid stage were observed and recorded. Nematodes were considered dead when no response to gentle touch with a platinum wire was detected.

Body length and body width measurement: Synchronized L1-stage nematodes were transferred to treatment plates and cultured until the gravid adult stage, followed by washing with M9 buffer. The nematodes were mounted on 2% agarose pads, anesthetized, and photographed using a stereomicroscope. Body length (maximum longitudinal axis) and body width (transverse distance in the widest abdominal region) were measured using ImageJ 1.4q. At least 30 nematodes were randomly measured per group, and all experiments were independently repeated in triplicate [28].

#### 2.4.4. Measurement of Physical Activity Indicators

Head thrust frequency: Nematodes treated to the young adult stage were transferred to bacteria-free NGM plates, and a small volume of M9 buffer was added to form a liquid medium. The number of effective lateral head thrusts within 20 s for individual nematodes was recorded under the stereomicroscope (one valid thrust was defined as displacement exceeding 50% of the body length relative to the base of the pharyngeal pump). At least 30 nematodes were examined per group.

Pharyngeal pumping frequency: Nematodes treated to the gravid stage were transferred to fresh treatment plates and incubated for 1 h. The number of pharyngeal pumps within 20 s for individual nematodes was observed and recorded under the stereomicroscope. At least 30 nematodes were assayed per group.

#### 2.4.5. Measurement of Reproductive Capacity Indicators

Brood size determination: Gravid adult nematodes after treatment were rinsed with M9 buffer and placed on glass slides. A lysis solution containing NaOCl and NaOH was added with gentle agitation to dissolve the nematode body and release eggs. The number of eggs per individual nematode was counted directly under a microscope. At least 20 nematodes were examined per group.

Total progeny assay: Synchronized L1-stage nematodes were transferred to NGM plates supplemented with JPCP-70-0 and cultured to the gravid adult stage. Nematodes were transferred to fresh plates daily during the oviposition period. Once all eggs on the original plates hatched, the viable progeny were counted. At least 30 nematodes were tested per group [29].

### 2.5. Statistical Analysis

All experiments were performed independently in triplicate. Data were expressed as the mean ± standard deviation. Statistical analysis was conducted using GraphPad Prism 8 software. One-way analysis of variance was applied for comparisons among multiple groups, followed by Duncan’s test or Tamhane’s T2 test for post hoc analysis. A *p* * value less than 0.05 indicated a statistically significant difference.

## 3. Results

### 3.1. Structural Characterization

Polysaccharides can be classified into homopolysaccharides and heteropolysaccharides based on the differences in their monosaccharide composition. Among these, heteropolysaccharides are high-molecular-weight compounds formed by the linkage of various distinct monosaccharide units through glycosidic bonds [30]. Therefore, clarifying the chemical structure and related characteristics of polysaccharides is a prerequisite for the in-depth investigation of their biological activities. Currently, a series of modern biochemical detection techniques, such as GC-MS, FT-IR, NMR, and methylation analysis, have been widely applied for determining the molecular weight and structural features of polysaccharides [31].

#### 3.1.1. Isolation, Purification, and Determination of the Basic Components of JPCP-70-0

The structural integrity and biological activity of polysaccharides were preserved to the greatest extent by employing freeze-drying to remove moisture from fresh JHPCH. Crude polysaccharides (designated JPCPs) were subsequently obtained via hot water extraction followed by a series of purification procedures, with a yield of 10.23%. Polysaccharides with distinct structures and molecular weights exhibited different solubilities in ethanol solutions of varying concentrations. Fractional ethanol precipitation was therefore adopted for the preliminary fractionation of polysaccharide fractions. In this study, ethanol solutions at concentrations of 20%, 50%, and 70% were sequentially applied to precipitate polysaccharide fractions of different polarities, which were designated JPCP-20, JPCP-50, and JPCP-70, respectively. The yields of the three fractions were calculated as 0.14%, 0.73%, and 3.67%, respectively (relative to the mass of JHPCH). JPCP-70 displayed a higher yield than JPCP-20 and JPCP-50, and was therefore selected for further purification and investigation.

**Figure 1:**
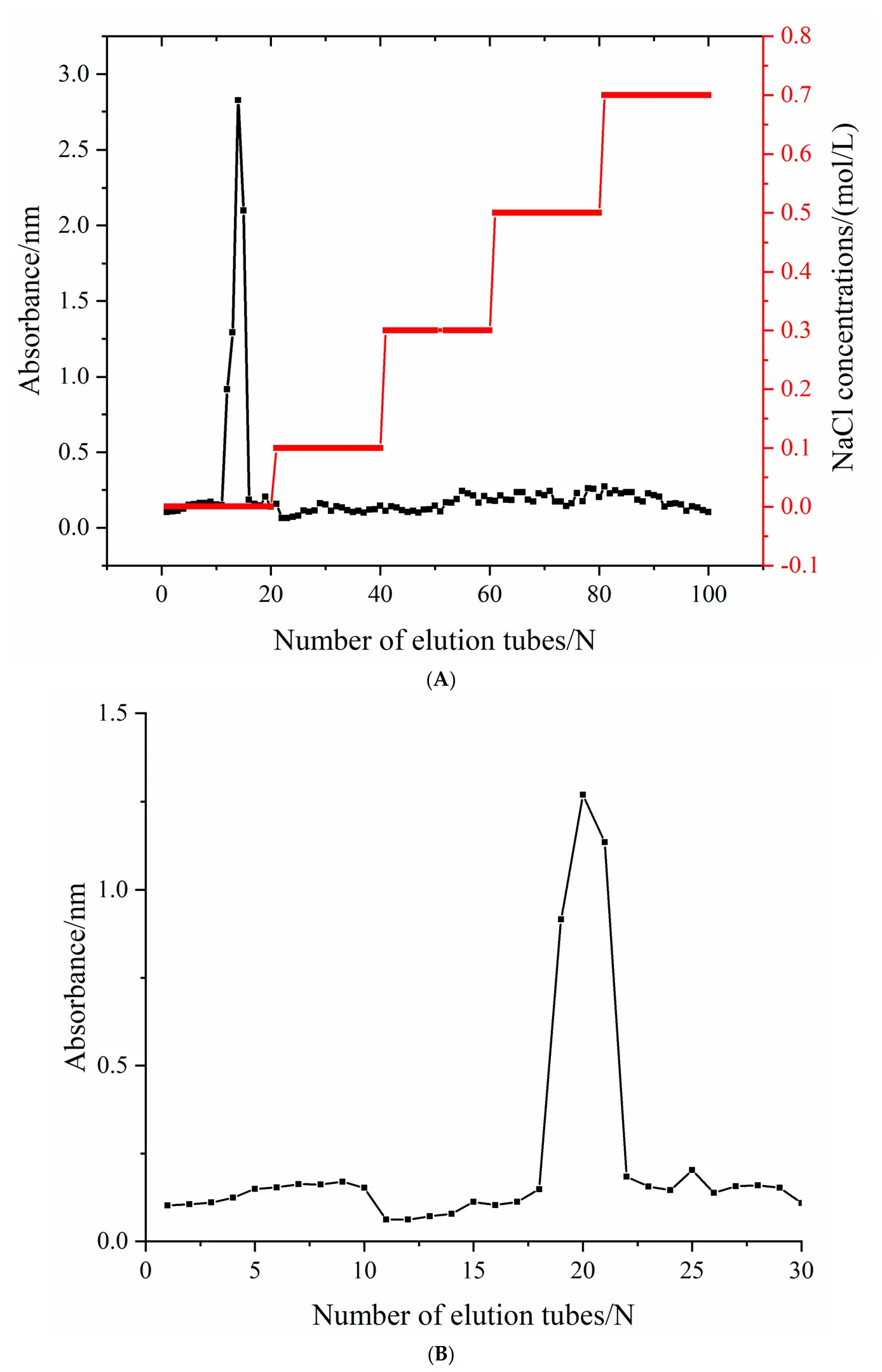

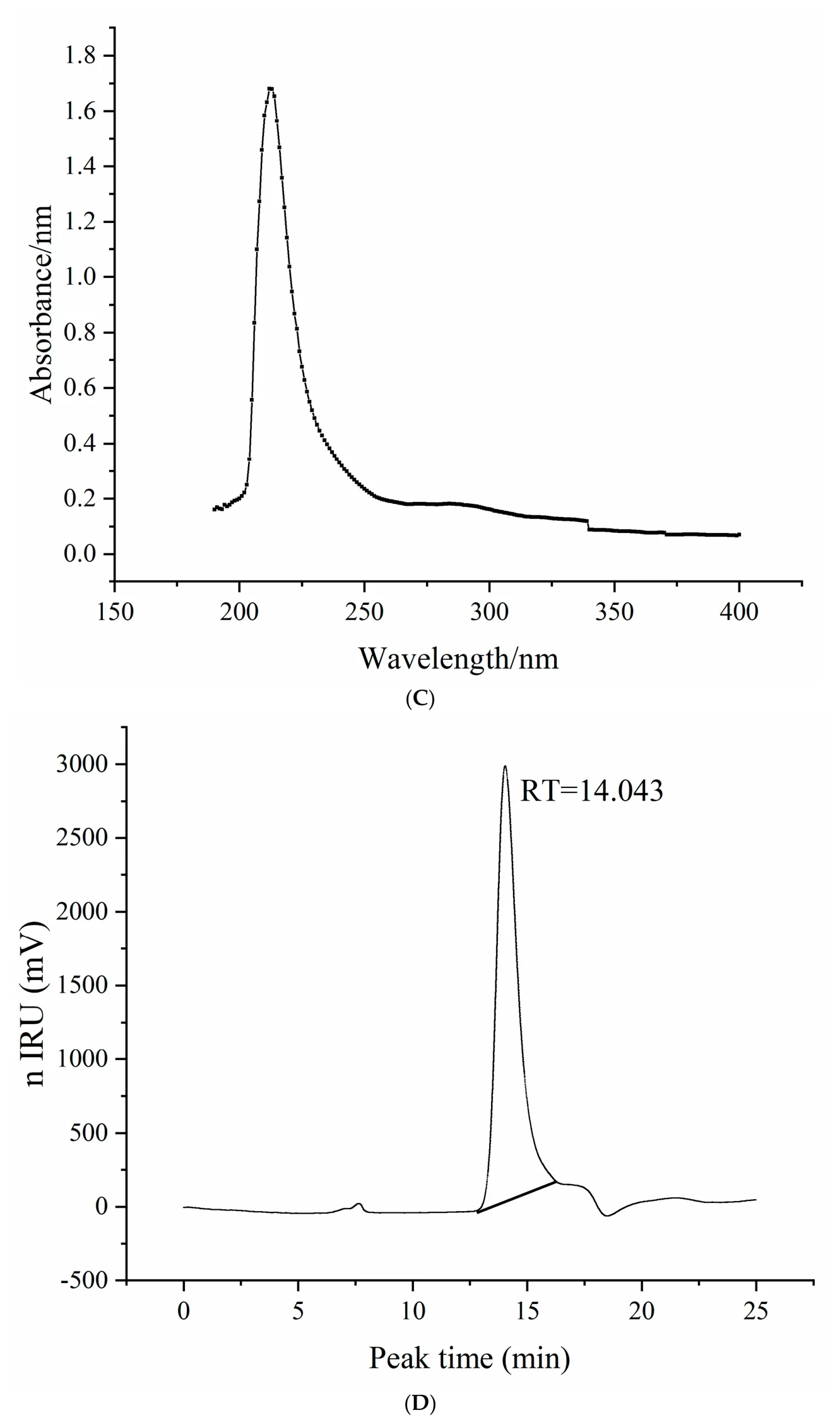
Isolation, purification and ultraviolet spectrum of JPCP-70-0. (**A**)—DEAE-52 cellulose column; (**B**)—Sephadex G-100; (**C**)—UV Spectrum; (**D**)—HPGPC chromatograms of JPCP-70-0.

Homogeneous polysaccharides cannot be acquired by fractional ethanol precipitation alone. Consequently, column chromatography was performed for further separation and purification. As illustrated in Figure 1A, JPCP-70 was fractionated using a DEAE-52 anion-exchange column. The fraction eluted with 0 mol/L NaCl, which exhibited the highest peak area, was collected and dialyzed for 72 h (3500 Da). After lyophilization, the fraction was loaded onto a Sephadex G-100 column, yielding a single symmetric peak (Figure 1B), indicative of high homogeneity. This purified polysaccharide fraction was designated JPCP-70-0, with a recovery rate of 14.26%.

The total carbohydrate content of JPCP-70-0 was determined to be 93.57% by the phenol–sulfuric acid method, confirming a high polysaccharide purity. The protein content was measured as 0.13% using the Coomassie brilliant blue method, demonstrating an extremely low residual protein level and efficient deproteinization. The uronic acid content was found to be 0.01% via the *m*-hydroxybiphenyl colorimetric assay, indicating JPCP-70-0 as a neutral polysaccharide, which was consistent with the finding that the fractions eluted with 0 mol/L NaCl were mostly neutral polysaccharides.

Ultraviolet-visible spectroscopic analysis (190–400 nm, Figure 1C) revealed no obvious absorption peaks at the characteristic wavelengths of 260 and 280 nm for JPCP-70-0, suggesting negligible amounts of protein and nucleic acid contaminants. This observation was in good agreement with the results obtained from the proximate chemical composition analysis.

#### 3.1.2. Determination of the Average Molecular Weight of JPCP-70-0

As shown in Figure 1D, a single, symmetric and sharp peak was observed in the HPLC chromatogram of JPCP-70-0, indicating its high purity. A calibration curve was established based on the elution times of dextran standards: lgMw = −0.3501t + 9.3765 (Figure S1), with a determination coefficient (R^2^) of 0.9795. The average relative molecular weight (Mw) of JPCP-70-0 was calculated to be 2.88 × 10^4^ Da from its retention time of 14.043 min.

#### 3.1.3. FT-IR Spectroscopic Analysis

**Figure 2:**
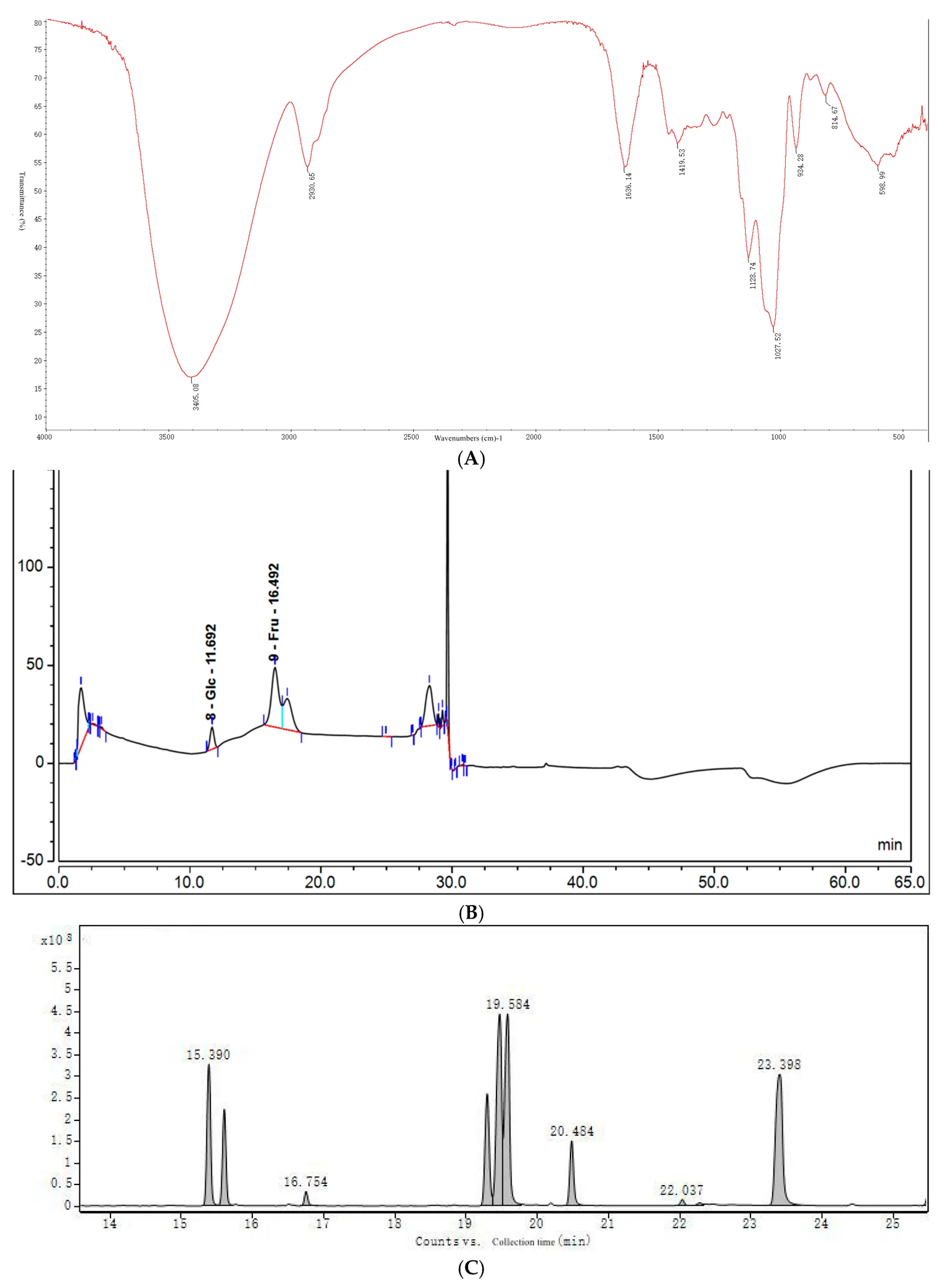
FT-IR spectrum (**A**), Ion chromatogram (**B**) and Total Ion Chromatogram of Methylation Products (**C**) of JPCP-70-0.

FT-IR spectroscopy is a well-established technique for identifying the functional groups in molecular structures and has been widely applied in the primary structural characterization of polysaccharides [14]. The FT-IR spectrum of JPCP-70-0 is presented in Figure 2A. The absorption band at 3405.08 cm^−1^ was assigned to the O–H stretching vibration, and the peak at 2930.65 cm^−1^ was attributed to the C–H stretching vibration. These two bands are characteristic of polysaccharides [26], confirming the presence of a typical polysaccharide structure in JPCP-70-0. The signal at 1636.14 cm^−1^ was ascribed to the C=O stretching vibration (carboxyl group) or the presence of bound water [32]. The peak at 1419.52 cm^−1^ was attributed to the C–H deformation vibration, whereas the peak at 1027.52 cm^−1^ was caused by the stretching vibrations of C–O–C in the pyranose ring, indicating that JPCP-70-0 possessed a pyranose ring structure [15]. The peaks at approximately 934.28 cm^−1^ were indicative of β-glycosidic linkages [16,24], suggesting that JPCP-70-0 was dominated by the β-configuration, which was consistent with the subsequent NMR results.

#### 3.1.4. Monosaccharide Composition Analysis of JPCP-70-0

The physiological functions of polysaccharides are related to their monosaccharide composition, molar ratios, and linkage sequences. Determination of the monosaccharide composition of JPCP-70-0 is beneficial for exploring its structure–activity relationship. The ion chromatography profile of monosaccharide composition in JPCP-70-0 is shown in Figure 2B (the chromatogram of mixed monosaccharide standards is presented in Figure S2. Chromatographic analysis revealed that JPCP-70-0 comprised two monosaccharides, namely glucose (Glc) and fructose (Fru). The molar ratio of the monosaccharide components was determined to be Glc:Fru = 6.65%:93.35%. Notably, significant variations in monosaccharide composition can be observed for polysaccharides derived from different sources of *Polygonatum cyrtonema*. Wang et al. [33] found that the purified polysaccharide fractions isolated from *Polygonatum cyrtonema* were composed of galactose, rhamnose, mannose, glucose and xylose. Wang et al. [34] further reported that polysaccharides from *Polygonatum cyrtonema* Hua were mainly composed of glucose and mannose. Such discrepancies in monosaccharide composition may originate from differences in raw-material origin, processing methods, and extraction and purification procedures.

#### 3.1.5. Methylation Analysis of JPCP-70-0

Methylation analysis is a widely used method for determining glycosidic linkages, by which the linkage patterns of monosaccharide residues in polysaccharides are identified. Free hydroxyl groups in the polysaccharide were methylated, and the methylated products were subsequently hydrolyzed, reduced, and derivatized. The partially methylated alditol acetates (PMAAs) were analyzed using GC-MS to determine the types of glycosidic linkages.

The total ion chromatogram of methylation analysis for JPCP-70-0 is displayed in Figure 2C, revealing nine distinct signal peaks. The linkage patterns of individual peaks and fragments derived from partially methylated alditol acetates of JPCP-70-0 were identified by MS combined with the relative retention times in GC. The percentages of methylated residues were estimated by calculating the peak area ratios, with the results presented in Table 1. As shown in the table, JPCP-70-0 mainly comprised Fru*f* -(2→, Glc*p*-(1→, →6)-Fru*f*-(2→, →1)-Fru*f*-(2→, →6)-Glc*p*-(1→, and →1,6)-Fru*f*-(2→, with the corresponding proportions of 20.85%, 1.25%, 15.82%, 37.45%, 5.61%, and 19.02%, respectively. The molar ratio of Fru to Glc determined by methylation analysis was 93.18%:6.86%, suggesting that the main chain of JPCP-70-0 was predominantly composed of fructose and glucose residues. This result was consistent with that obtained from monosaccharide composition analysis.

#### 3.1.6. NMR Spectroscopic Analysis of JPCP-70-0

**Figure 3:**
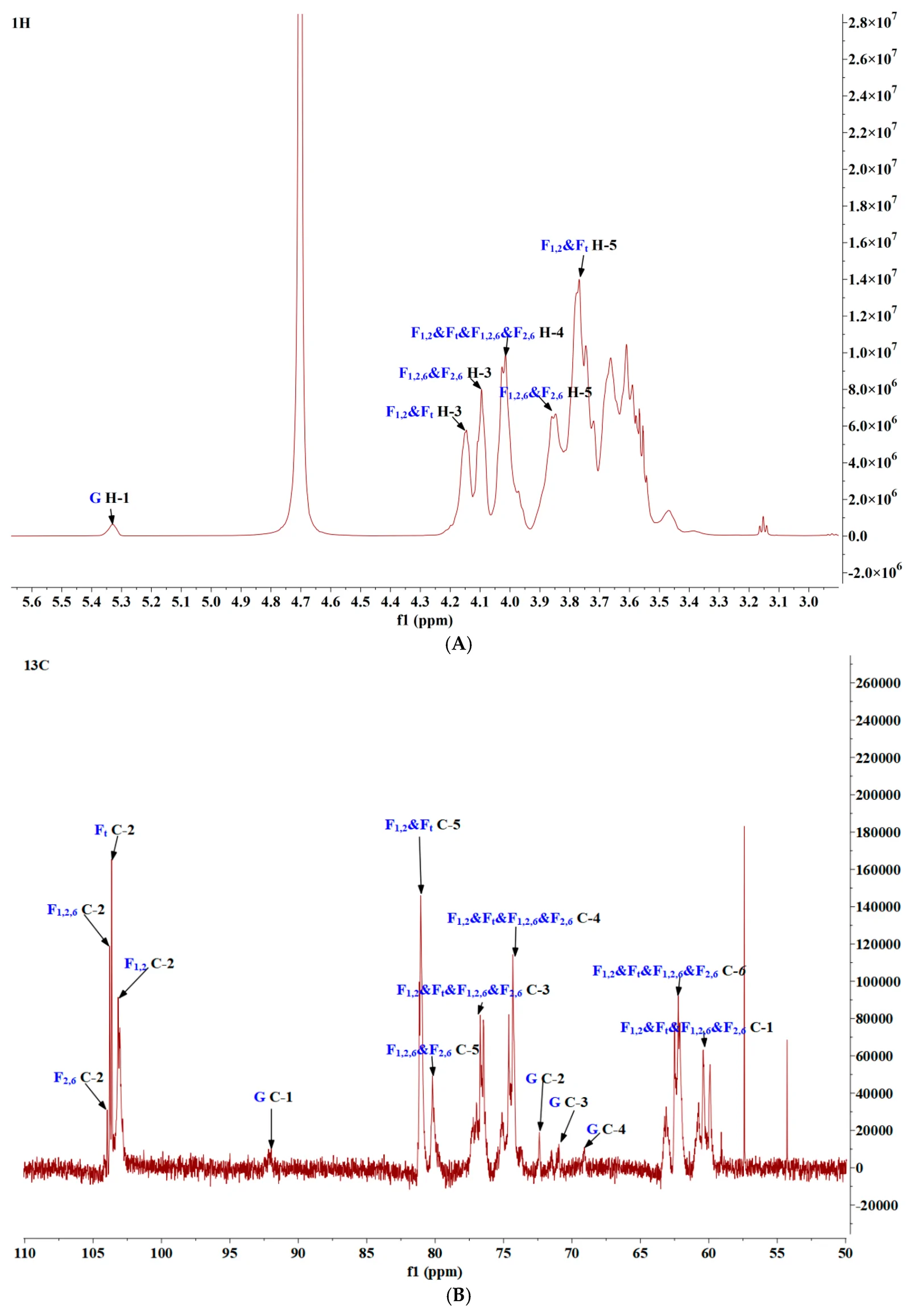

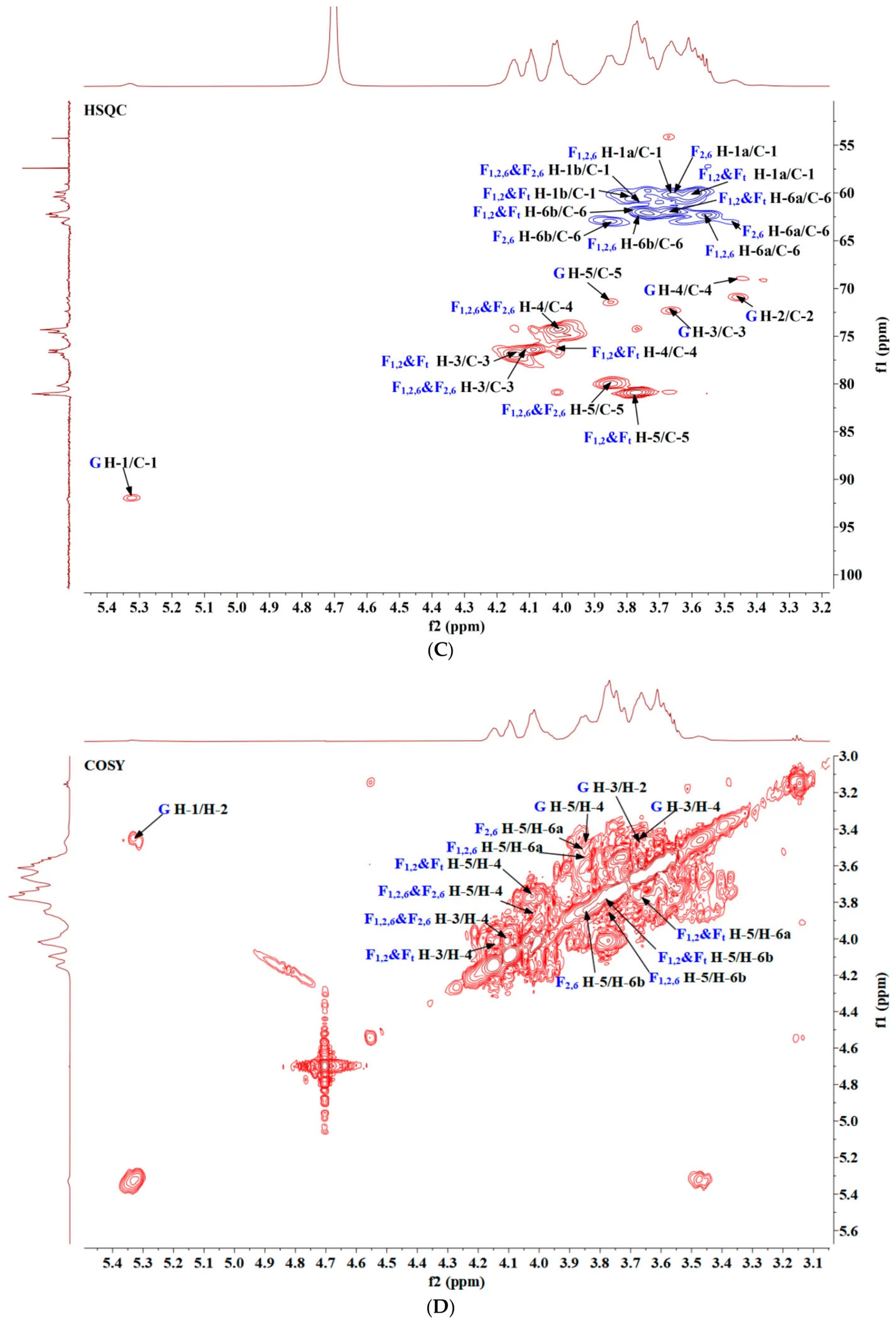

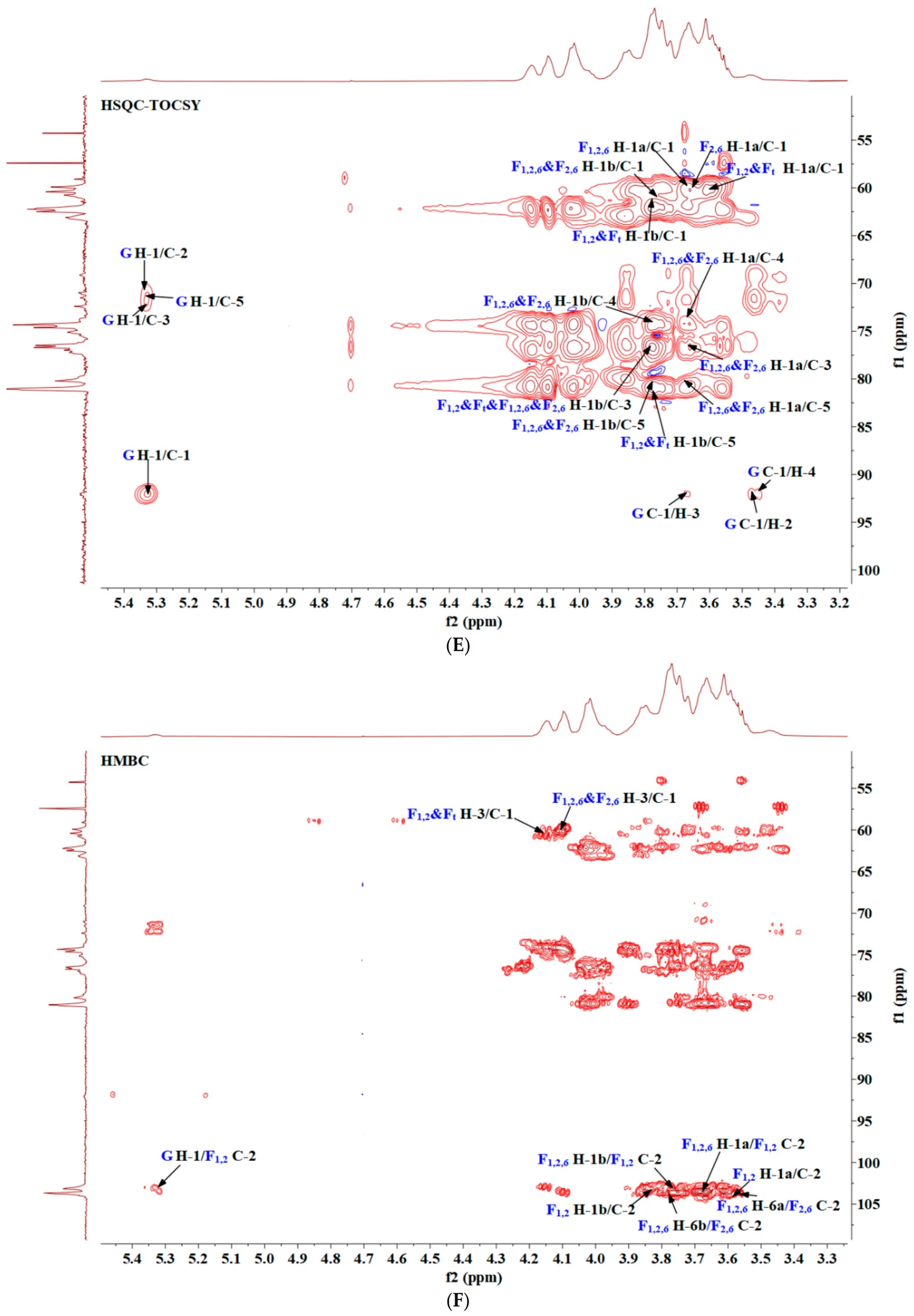

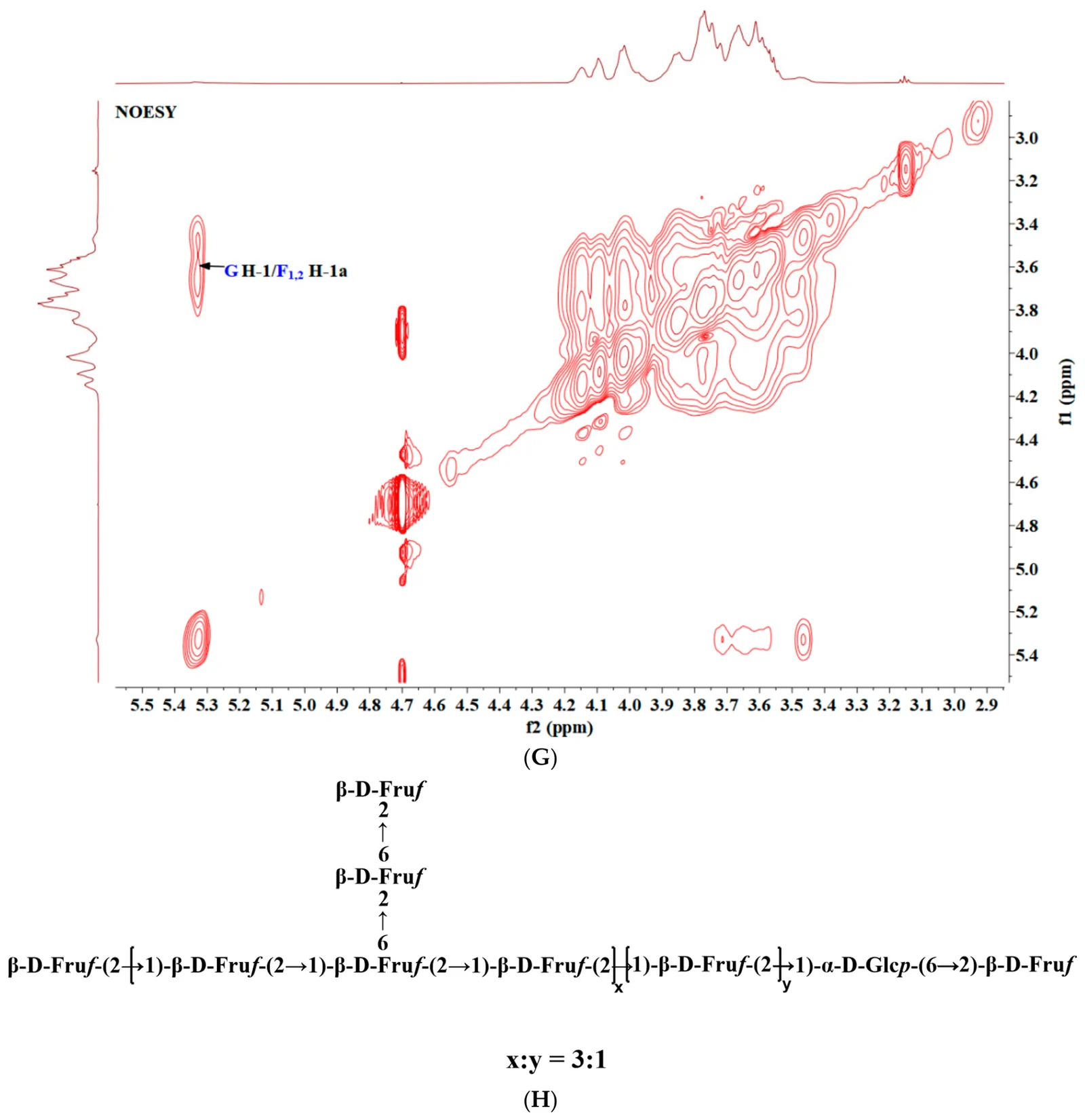
1D/2D NMR spectrum of JPCP-70-0 (600 MHz, solvent D2O). (**A**): **^1^**H, (**B**): ^13^C, (**C**):HSQC, (**D**): COSY, (**E**): HSQC-TOCSY, (**F**): HMBC, (**G**): NOESY, (**H**): Structural Drawing of JPCP-70-0.

Comprehensive elucidation of the polysaccharide chain structure required the application of 1D/2D NMR spectroscopy, combined with the results of monosaccharide composition and methylation analysis, to deduce the complete structure of the polysaccharide chain. As shown in Figure 3B, two sets of signals were observed in the anomeric carbon region at *δ* 90–110 in the ^13^C NMR spectrum of JPCP-70-0, indicating the presence of two monosaccharides. Monosaccharide composition and methylation analysis demonstrated that JPCP-70-0 mainly comprised fructose. Since ketose lacks anomeric protons, no corresponding anomeric proton signals were detected in the ^1^H-NMR spectrum (Figure 3A) [35]. Meanwhile, the anomeric carbon signals at *δ* 103.65 ppm, *δ* 103.16 ppm, *δ* 103.79 ppm, and *δ* 103.98 ppm were consistent with the characteristic NMR signals of fructose residues. These signals were assigned to the anomeric carbon (C-2) of furan-type fructose residues. The methylation analysis and previously reported literature [35,36,37,38,39] indicated that these signals were, respectively, attributed to the anomeric carbon C-2 of →1)-β-D-Fru*f*-(2→, β-D-Fru*f*-(2→, →1,6)-β-D-Fru*f*-(2→, and →6)-β-D-Fru*f*-(2→, which were sequentially designated as fructose residues F_1,2_, F_t_, F_1,2,6_, and F_2,6_.

The signal at *δ* 5.32 ppm in the ^1^H NMR spectrum was attributed to the anomeric proton of Glcp. A cross-peak between this proton signal and the signal at *δ* 92.11 ppm was observed in both the HSQC spectrum (Figure 3C) and the HSQC–TOCSY spectrum (Figure 3E). Based on this correlation, the sugar residue was deduced to be →6)-α-D-Glc*p*-(1→ and designated as G, which was proposed to be 6-O-substituted by β-D-Fru in the form of a neoketose linkage [38,39]. Partial proton cross-peaks related to →6)-α-D-Glc*p*-(1→ were also detected in the ^1^H-^1^H COSY spectrum (Figure 3D). According to ^1^H NMR, ^13^C NMR, and HSQC spectra, combined with previous reports, the chemical shift values of ^1^H and ^13^C signals for the sugar residues F_1,2_, F_t_, F_1,2,6_, and F_2,6_ and G were determined and are listed in Table 2.

HMBC (Figure 3F) and NOESY (Figure 3G) spectra revealed several cross-peaks representing the correlations between ^1^H and ^13^C signals from different sugar residues.

A cross-peak between H-1 of residue G (δ 5.32 ppm) and C-2 of residue F_1,2_ (δ 103.65 ppm) was observed (G H-1/F_1,2_ C-2). Moreover, a cross-peak between H-1 of residue G (δ 5.32 ppm) and H-1a of residue F_1,2_ (δ 3.61 ppm) was detected in the NOESY spectrum (G H-1/F_1,2_ H-1a). Given the lack of anomeric proton signals in the ^1^H-NMR spectrum of fructans, the HMBC correlation involving H-1 of the adjacent →1)-β-D-Fru*f*-(2→ residue indicated the presence of the linkage sequence →1)-β-D-Fru*f*-(2→1)-α-D-Glc*p*-(6→, with the glycosidic bond formed at the O-2 position.

Cross-peaks were observed between H-1a of residue F_1,2,6_ (*δ* 3.66 ppm) and C-2 of residue F_1,2_ (*δ* 103.65 ppm) (F_1,2,6_ H-1a/F_1,2_ C-2), as well as between H-1b of residue F_1,2,6_ (*δ* 3.77 ppm) and C-2 of residue F_1,2_ (*δ* 103.65 ppm) (F_1,2,6_ H-1b/F_1,2_ C-2). These correlations indicated a β(2→1) linkage between →1)-β-D-Fru*f*-(2→ and →1,6)-β-D-Fru*f*-(2→, with the connection occurring at the O-2 position.

A cross-peak between H-1a of residue F_1,2_ (*δ* 3.61 ppm) and C-2 of residue F_1,2,6_ (*δ* 103.79 ppm) (F_1,2_ H-1a/F_1,2,6_ C-2) was detected, suggesting a β(2→1) linkage between →1,6)-β-D-Fru*f*-(2→ and →1)-β-D-Fru*f*-(2→ at the O-2 position.

Cross-peaks were observed between H-1a of residue F_1,2_ (*δ* 3.61 ppm) and C-2 of residue F_1,2_ (δ 103.65 ppm) (F_1,2_ H-1a/F_1,2_ C-2), and between H-1b of residue F_1,2_ (*δ* 3.77 ppm) and C-2 of residue F_1,2_ (*δ* 103.65 ppm) (F_1,2_ H-1b/F_1,2_ C-2). These results confirmed the presence of a β(2→1) linkage within the sequence →1)-β-D-Fru*f*-(2→1)-β-D-Fru*f*-(2→ at the O-2 position.

Cross-peaks were detected between H-6a of residue F_1,2,6_ (*δ* 3.56 ppm) and C-2 of residue F_2,6_ (*δ* 103.98 ppm) (F_1,2,6_ H-6a/F_2,6_ C-2), and between H-6b of residue F_1,2,6_ (δ 3.77 ppm) and C-2 of residue F_2,6_ (*δ* 103.98 ppm) (F_1,2,6_ H-6b/F_2,6_ C-2). These correlations indicated a β(2→6) linkage between →6)-β-D-Fru*f*-(2→ and →1,6)-β-D-Fru*f*-(2→ at the O-6 position.

Based on the methylation analysis, the molar ratio of sugar residues F_1,2_, F_t_, F_1,2,6_, F_2,6_, and G was calculated as (21.97 + 15.48):(12.76 + 8.09):19.02:(10.75 + 5.07):5.61 = 37.45:20.85:19.02:15.82:5.61 = 6.68:3.72:3.39:2.82:1.00. The molar ratio of fructose residues F_1,2_, F_1,2,6,_ and F_2,6_ was approximately 7:3:3.

Based on the combined results of 1D/2D NMR, monosaccharide composition, FT-IR, and methylation analysis, the backbone structure of JPCP-70-0 was finally deduced to be a fructan, with a main chain composed of →1)-β-D-Fru*f*-(2→ residues and side chains composed of →6)-β-D-Fru*f*-(2→ residues, as illustrated in Figure 3H.

### 3.2. Effects of JPCP-70-0 on Health Parameters in C. elegans

#### 3.2.1. Effects of JPCP-70-0 on Survival, Development, and Body Size in *C. elegans*

**Figure 4:**
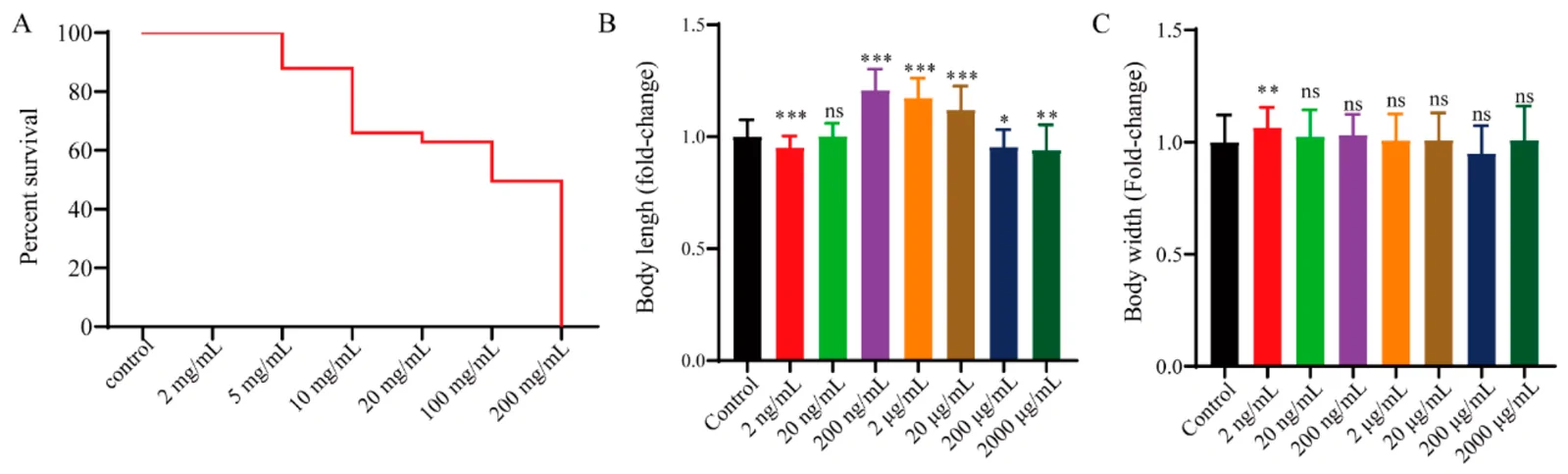
Effects of JPCP-70-0 on survival (**A**), body length (**B**), and body width (**C**) of *C. elegans*. *, *p* < 0.05; **, *p* < 0.01; ***, *p* < 0.001.

This manuscript investigated the potential effects of JPCP-70-0 on the physiological functions of *C. elegans* as a model organism by evaluating the tolerance of nematodes to various concentrations of JPCP-70-0 and analyzing its influences on indices related to growth and development. As shown in Figure 4A, treatment with JPCP-70-0 at different concentrations (0–200 mg/mL) resulted in concentration-dependent changes in the growth, development, and survival rate of nematodes.

The developmental process of nematodes in the 2 and 5 mg/mL treatment groups was generally consistent with that in the control group; the survival rates in the egg-bearing stage were recorded as 100% and 87.91%, respectively. In contrast, developmental delay was observed in the 10, 20, and 100 mg/mL groups. The inhibitory effect was enhanced with increasing concentration, accompanied by a prolonged time required to reach the egg-bearing stage. The 200 mg/mL treatment group exhibited the most severe developmental delay; all individuals died before the L3 stage.

The effects of JPCP-70-0 on growth and development were further quantified using body length and body width as indices. As shown in Figure 4B, the influence of JPCP-70-0 on body length exhibited an inverted U-shaped trend. The body length was significantly reduced to 95.17% of that in the control group at 2 ng/mL. The body length was gradually elevated with increasing concentrations, with significant increases of 20.86%, 17.33%, and 12.04% observed at 200 ng/mL, 2 μg/mL, and 20 μg/mL, respectively. However, the body length was decreased again to 95.47% and 94.98% of the control value, respectively, at higher concentrations of 200 μg/mL and 2000 μg/mL.

A significant increase in body width (6.44% compared with the control) was detected only in the 2 ng/mL treatment group, with no obvious changes observed in the other concentration groups (Figure 4C).

In summary, the present study preliminarily demonstrated that JPCP-70-0 mildly impacted the survival and development of *C. elegans* at relatively low concentrations, whereas obvious developmental inhibition and lethal effects were observed at higher concentrations. The non-monotonic, concentration-dependent changes in body length suggested that JPCP-70-0 might regulate the growth process of nematodes within a certain concentration range.

#### 3.2.2. Effects of JPCP-70-0 on Locomotion in *C. elegans*

**Figure 5:**
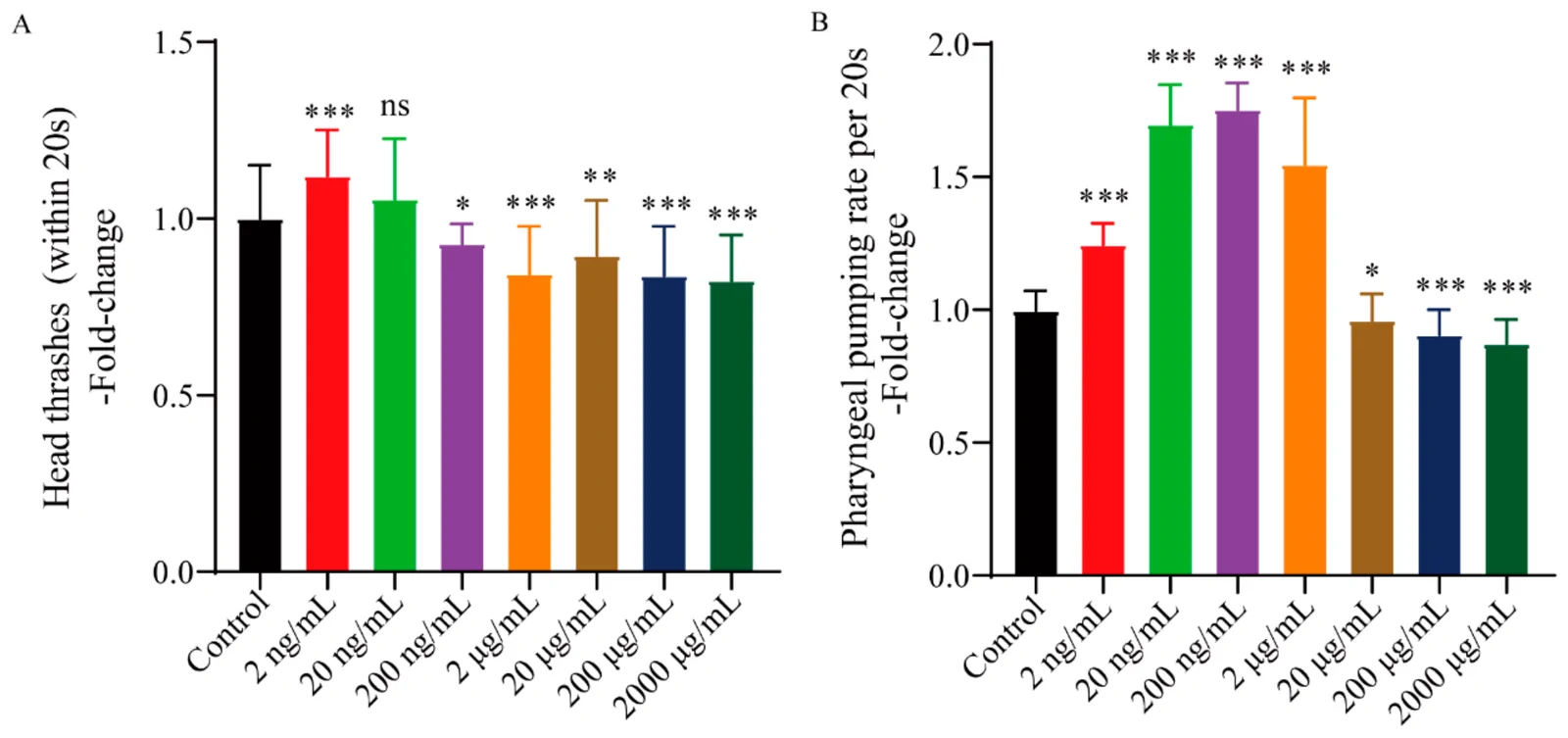
Effects of JPCP-70-0 on locomotor ability of *C. elegans*. (**A**). 70. (**B**). Changes in pharyngeal pumping rate of *C. elegans* after treatment with JPCP-70-0. Significant differences compared with the control group are the same as described above. *, *p* < 0.05; **, *p* < 0.01; ***, *p* < 0.001.

The head thrashing frequency (reflecting locomotor activity) and pharyngeal pumping rate (reflecting feeding behavior) were selected as key indicators to evaluate the potential effects of JPCP-70-0 on the locomotor behavior of *Caenorhabditis elegans*. Also, the behavioral changes in nematodes treated with various concentrations of JPCP-70-0 were determined. The results are presented in Figure 5.

Head thrashing frequency was altered in a dose-dependent manner (Figure 5A). The head thrashing frequency significantly increased by 12.10% in the 2 ng/mL treatment group compared with the control group, with no significant difference observed in the 20 ng/mL group. When the concentration was elevated to 200 ng/mL and above, head thrashing was markedly inhibited, with significant reductions of 7.07%, 15.66%, 10.48%, 16.13%, and 17.58% detected in the respective treatment groups compared with the control group.

Pharyngeal pumping rate was more sensitive to JPCP-70-0 treatment and exhibited an obvious biphasic effect (Figure 5B). Within the concentration range of 2–2000 ng/mL, JPCP-70-0 significantly promoted pharyngeal pumping activity, with the frequency increased by 24.47%, 69.86%, 75.37%, and 54.70% compared with that in the control group, respectively. However, pharyngeal pumping was inhibited at higher concentrations (20–2000 μg/mL) and decreased in a concentration-dependent manner, with reductions of 3.95%, 9.44%, and 12.75% compared with the control, respectively.

The aforementioned results indicated that JPCP-70-0 regulated the locomotor behavior of *C. elegans* in a concentration-dependent manner. Low concentrations (e.g., 2 ng/mL) could stimulate locomotor and feeding activities to a certain extent, whereas high concentrations generally exerted inhibitory effects. Moreover, pharyngeal pumping behavior was more sensitive to JPCP-70-0 treatment than head thrashing.

#### 3.2.3. Effects of JPCP-70-0 on Reproduction in *C. elegans*

**Figure 6:**
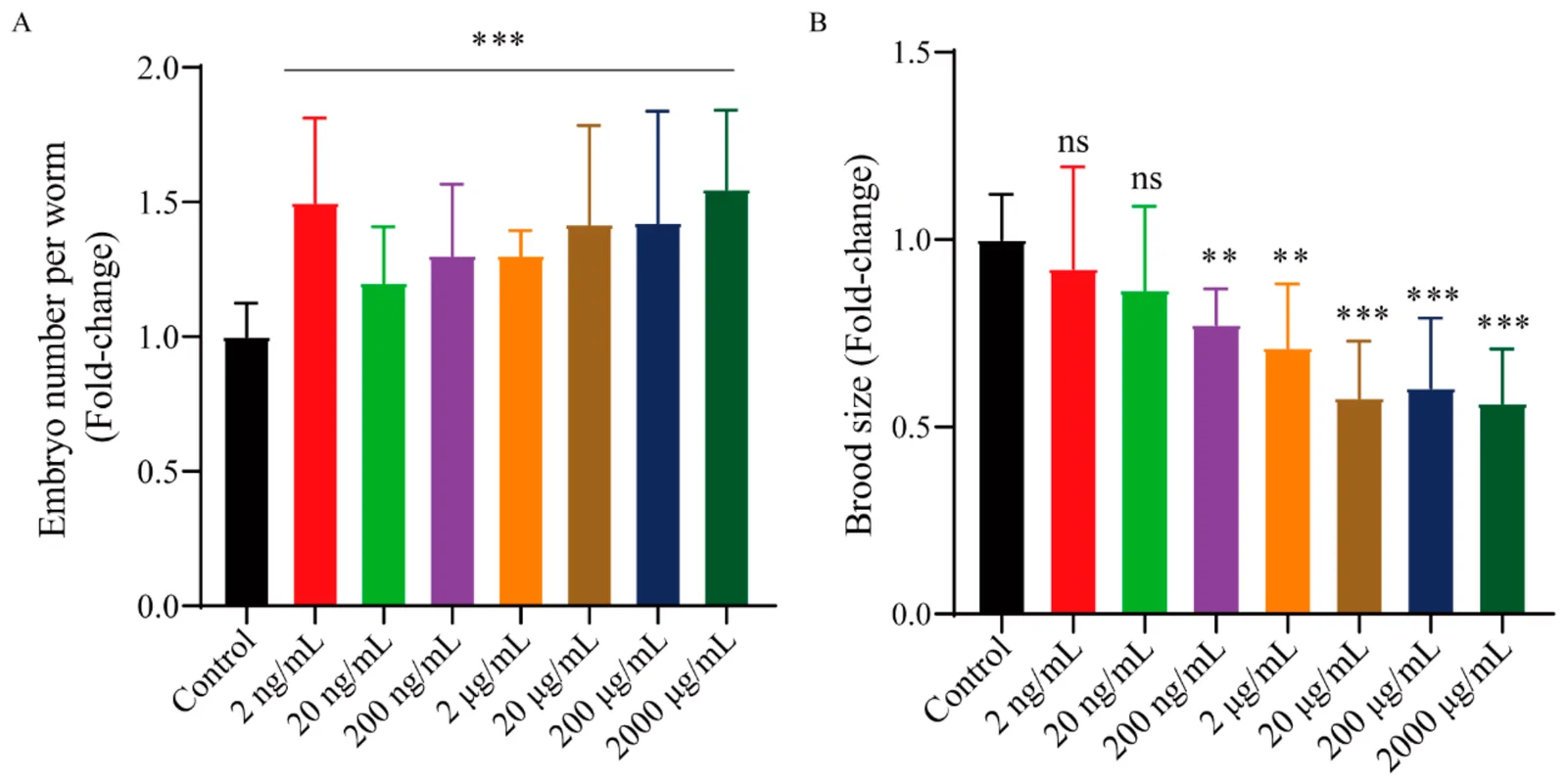
Effects of JPCP-70-0 on reproductive capacity of *C. elegans*. (**A**) Changes in the number of eggs in utero of *C. elegans* after treatment with JPCP-70-0; (**B**) Changes in the number of progeny of *C. elegans* after treatment with JPCP-70-0. Significant differences compared with the control group are the same as described above. **, *p* < 0.01; ***, *p* < 0.001.

This manuscript investigated the impact of JPCP-70-0 on the reproductive function of *C. elegans* by determining the number of eggs in utero and total progeny of nematodes treated with different concentrations of JPCP-70-0 to evaluate its overall effect on reproductive output. The results are shown in Figure 6.

The number of eggs in utero of nematodes significantly increased under JPCP-70-0 treatment at all tested concentrations; each treatment group showed an increase of more than 20% compared with the control group (Figure 6A). However, a concentration-dependent inhibitory effect was observed on the total number of progeny (Figure 6B): low-concentration treatments (2 and 20 ng/mL) had no significant effect on the number of progeny. However, when the concentration reached 200 ng/mL and above, the total number of progeny significantly decreased and the inhibitory effect was enhanced with the increase in concentration, showing a reduction of 22.72–43.64% compared with that in the control group.

The aforementioned results indicated that the number of eggs in utero of *C. elegans* significantly increased under JPCP-70-0 treatment, but the final number of progeny was simultaneously reduced at higher concentrations. This suggested the impact of JPCP-70-0 treatment on egg development, hatching, or early survival, thereby exhibiting a concentration-dependent inhibitory effect on overall reproductive output. This might be caused by abnormal egg development, decreased larval viability, or disturbed nutrient supply during larval development induced by high concentrations of polysaccharides [18,39].

## 4. Conclusions

(1) The crude polysaccharide JPCP extracted from JHPCP by water extraction and ethanol precipitation was isolated and purified using DEAE-52 and Sephadex G-100 chromatography. The purified homogeneous polysaccharide was designated JPCP-70-0, with a total sugar content of 93.57%. The results of monosaccharide composition and molecular weight analysis revealed that JPCP-70-0 was a neutral fructan with a molecular weight of 2.88 × 10^4^ Da; it mainly comprised fructose (93.35%) and glucose (6.65%), with almost no protein or uronic acid.

(2) FT-IR indicated that JPCP-70-0 had characteristic polysaccharide functional groups such as O–H, C–H, and C–O–C. Moreover, pyranose ring structures were observed, together with both α- and β-glycosidic linkages, among which the abundance of β-glycosidic linkages was high.

(3) Methylation and 1D/2D NMR analysis demonstrated that the backbone of JPCP-70-0 was mainly formed by →1)-β-D-Fru*f*-(2→ residues, with →6)-β-D-Fru*f*-(2→ residues serving as side chains. The molar ratio of the key fructose residues (F_1,2_; F_1,2,6_; F_2,6_) was determined to be approximately 7:3:3. Moreover, →6)-α-D-Glc*p*-(1→ residues were detected, which were linked to β-D-fructose at the O-2 position to form difructose anhydride-like connections.

(4) The present study systematically investigated the regulatory effects of JPCP-70-0 on the growth and development, body morphology, locomotor behavior, feeding activity, and reproductive capacity of *C. elegans*. The results showed that the growth and development of nematodes exhibited a concentration-dependent regulatory effect on JPCP-70-0 treatment. Low concentrations had negligible impacts on nematode survival and development, but they promoted growth, stimulated locomotor and feeding activities, and increased the number of eggs in utero. In contrast, high concentrations induced developmental inhibition, decreased locomotor ability, and reduced reproductive output.

## Figures and Tables

**Table 1:** Results of the methylation analysis of JPCP-70-0.

PMAAs	Linkage	RT (min)	Major Mass Fragments (*m*/*z*)	Molar Ratios (%)
2,5-di-O-acetyl-1,3,4,6-tetra-O-methyl mannitol	Fru*f*-(2→	15.39	75, 87, 101, 129, 145, 161, 162, 205	12.76
2,5-di-O-acetyl-1,3,4,6-tetra-O-methyl glucitol	Fru*f*-(2→	15.606	75, 87, 101, 129, 146, 161, 162, 206	8.09
1,5-di-O-acetyl-2,3,4,6-tetra-O-methyl glucitol	Glc*p*-(1→	16.754	71, 87, 101, 102, 118, 129, 145, 161, 162, 205	1.25
2,5,6-tri-O-acetyl-1,3,4-tri-O-methyl mannitol	→6)-Fru*f*-(2→	19.294	72, 87, 99, 99, 129, 146, 162, 189	10.75
1,2,5-tri-O-acetyl-3,4,6-tri-O-methyl mannitol	→1)-Fru*f*-(2→	19.469	71, 87, 100, 101, 113, 129, 145, 190	21.97
2,5,6-tri-O-acetyl-1,3,4-tri-O-methyl glucitol	→6)-Fru*f*-(2→	19.584	71, 87, 101, 102, 113, 118, 129, 130, 145, 146, 161, 162, 189, 190	5.07
1,2,5-tri-O-acetyl-3,4,6-tri-O-metyl glucitol	→1)-Fru*f*-(2→	19.584	71, 87, 100, 102, 118, 129, 162, 189, 233	15.48
1,5,6-tri-O-acetyl-2,3,4-tri-O-methyl gluctiol	→6)-Glc*p*-(1→	20.484	71, 87, 88, 99, 100, 129, 130, 189, 190	5.61
1,2,5,6-tetra -O-acetyl-3,4-di-O-methyl-hexitol (mannitol, glucitol)	→1,6)-Fru*f*-(2→	23.398	75, 87, 101, 129, 145, 161, 162, 205	19.02

**Table 2:** Chemical shifts of ^1^H and ^13^C of each sugar residue.

Glycosyl Residues		Chemical Shifts (ppm)					
		H1/C1	H2/C2	H3/C3	H4/C4	H5/C5	H6/C6
F_1,2_	→1)-β-D-Fru*f*-(2→	3.61/3.77	/	4.14	4.02	3.77	3.66/3.77
		60.41	103.65	76.64	76.51	81.05	62.1
F_t_	β-D-Fru*f*-(2→	3.6/3.76	/	4.14	4.02	3.77	3.66/3.76
		60.43	103.16	76.58	76.55	81.04	62.09
F_1,2,6_	→1,6)-β-D-Fru*f*-(2→	3.66/3.77	/	4.10	4.01	3.85	3.56/3.77
		60.31	103.79	76.38	74.34	80.21	62.41
F_2,6_	→6)-β-D-Fru*f*-(2→	3.64/3.76	/	4.10	4.01	3.85	3.49/3.85
		60.32	103.98	76.35	74.38	80.20	63.06
G	→6)-α-D-Glc*p*-(1→	5.32	3.46	3.66	3.45	3.85	3.7/3.94
		92.11	70.94	72.33	69.08	71.36	60.33

## Data Availability

The original contributions presented in the study are included in the article/Supplementary Materials, further inquiries can be directed to the corresponding author.

## Supplementary Materials

The following supporting information can be downloaded at https://www.mdpi.com/article/10.3390/molecules31193542/s1. Figure S1: Dextran standard curve. Figure S2: Chromatogram of mixed monosaccharide standards. Table S1: Elution parameters.
