# Supplementary material for: Polygonatum cyrtonema Hua in Jiuhua Mountain: Structural Characterization and Biological Activity
Source: Molecules. 2026 Oct 4;31(19):3542. doi: 10.3390/molecules31193542 (PMC13667172; doi:10.3390/molecules31193542)
Supplement: Supplementary file 1 [file molecules-31-03542-s001.zip › molecules-4555219-supplementary.pdf]

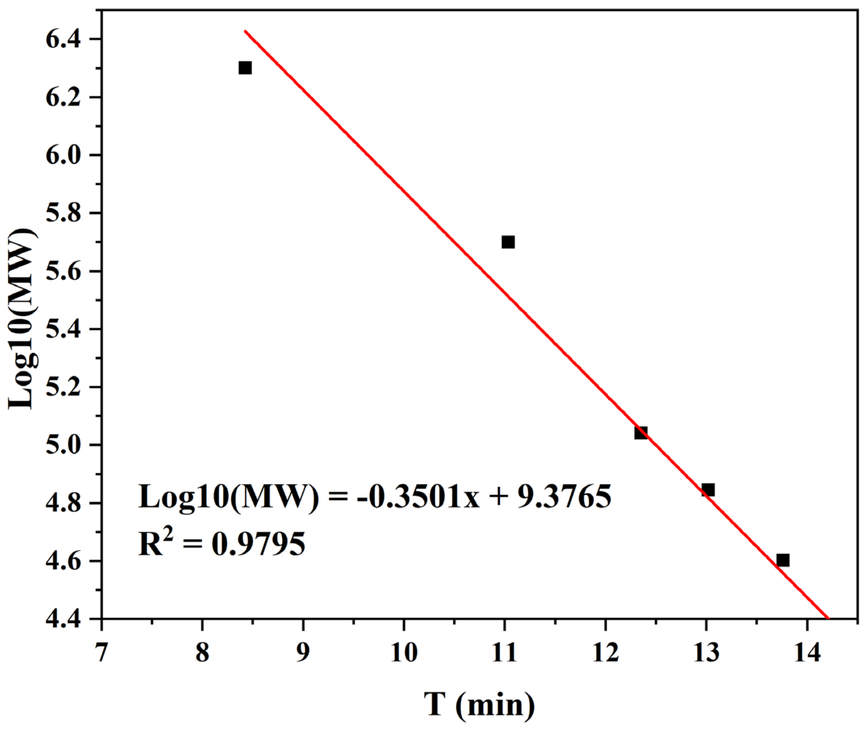

Fig. S1 Dextran standard curve

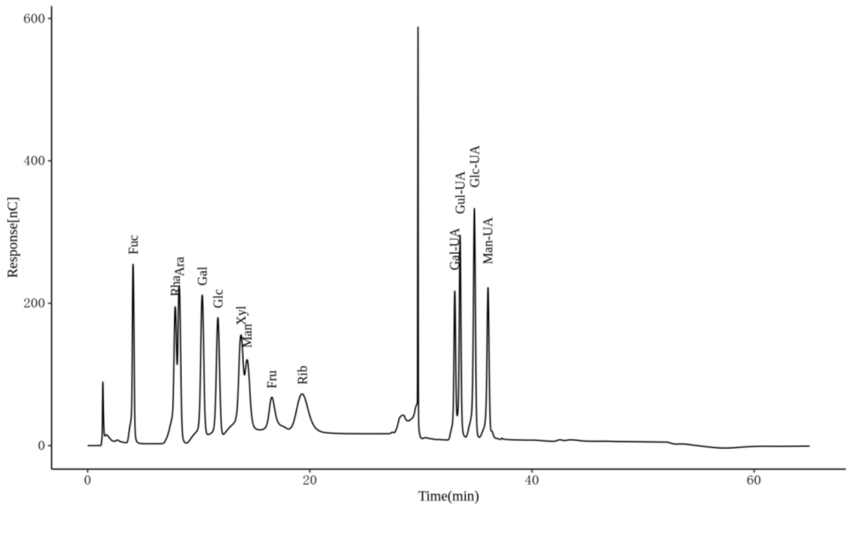

Fig. S2 Chromatogram of mixed monosaccharide standards

Table S1 Elution parameters

Time	Mobile phase (V/V)		
	A (H_2O)	B (0.1M NaOH)	C (0.1 M NaOH，0.2M NaAc)
0min	95	5	0
26min	85	5	10
42min	85	5	10
42.1min	60	0	40
52min	60	40	0
52.1min	95	5	0
60min	95	5	0
